# The association between the day of the week of milestones in the care pathway of patients with hip fracture and 30-day mortality: findings from a prospective national registry – The National Hip Fracture Database of England and Wales

**DOI:** 10.1186/s12916-017-0825-5

**Published:** 2017-03-27

**Authors:** Adrian Sayers, Michael R. Whitehouse, James R. Berstock, Karen A. Harding, Michael B. Kelly, Timothy J. Chesser

**Affiliations:** 1 Musculoskeletal Research Unit, School of Clinical Sciences, University of Bristol, Level 1 Learning and Research Building, Southmead Hospital, Westbury-on-Trym, Bristol, BS10 5NB UK; 2 School of Social and Community Medicine, University of Bristol, 39 Whatley Road, Bristol, BS8 2PS UK; 3 Avon Orthopaedic Centre, Brunel Building, Southmead Hospital, Westbury-on-Trym, Bristol, BS10 5NB UK

**Keywords:** Hip fracture, Mortality, Day of the week, Weekend effect, Admission, Surgery, Time to surgery, Discharge, Neck of femur fracture, Day of the week effects, National Hip Fracture Database, 30-day mortality

## Abstract

**Background:**

Recent publications indicate increased mortality in patients admitted to hospital at the weekend, but these findings may be subject to inadequate adjustment for case-mix and the complexities of resource provision. Hip fractures generally occur in a frail comorbid population with a consistent diagnosis precipitating admission as an emergency. We therefore aimed to examine the association between the day of the week of milestones in the care pathway and 30-day mortality in this population.

**Methods:**

Using data from a prospective national database of hip fractures, we investigated the association between day of the week of admission, surgery, inpatient stay, and discharge (care pathway milestones) and 30-day mortality using generalised linear models. Data was collected between January 1, 2011, and December 31, 2014, on 241,446 patients. An incremental case-mix adjustment strategy was performed using patient characteristics, non-surgical interventions, surgical interventions and discharge characteristics.

**Results:**

The day of admission was not associated with 30-day mortality. Sunday surgery (OR, 1.094; 95% CI, 1.043–1.148; *P* < 0.0001) and a delay to surgery of more than 24-hours (OR, 1.094; 95% CI, 1.059, 1.130; *P* < 0.0001) were both associated with a 9.4% increase in 30-day mortality. Discharge from the hospital on a Sunday (OR, 1.515; 95% CI, 1.224, 1.844; *P* < 0.0001) or out-of-hours discharge (OR, 1.174; 95% CI, 1.081, 1.276; *P* < 0.0001) were associated with a 51.5% and 17.4% increase in 30-day mortality, respectively. Mortality during the inpatient stay was 5.6% lower (IRR, 0.944; 95% CI, 0.909, 0.980; *P* = 0.003) at the weekend compared to weekdays.

**Conclusions:**

There is limited evidence of a generalised weekend effect in patients admitted to hospital for hip fracture. Optimising resource utilisation is an essential element of planning and delivering healthcare services. Interventions that lead to surgery within 24-hours of admission are justified. Factors such as Sunday operations, discharge and out-of-hours discharge require further investigation.

**Electronic supplementary material:**

The online version of this article (doi:10.1186/s12916-017-0825-5) contains supplementary material, which is available to authorized users.

## Background

There has been recent debate regarding the provision of healthcare across the week and whether there is an increase in mortality associated with admissions during the weekend, i.e. a ‘weekend effect’ [1–3]. Recent findings have not been universally accepted due to a perception of inadequate case mix adjustment and failure to consider the complexities of resource provision within each medical specialty in the statistical model [4]. Yet, this is not the first reported instance of a weekend effect, and there have been numerous publications across disciplines and healthcare settings reporting differential outcomes associated with the day of the week of admission [2, 3, 5, 6].

The current concept of a ‘weekend effect’ in healthcare is ill-defined, and current research typically focuses on one milestone in the patient care pathway, i.e. admission [1–3, 5–13]. However, the care pathway for acute medical problems will encompass multiple milestones that may extend across multiple days and weeks, and therefore include multiple week and weekend days. Given the complexities of analysing routinely collected clinical data, and the limitations of performing ‘one size fits all’ analyses across multiple specialties and healthcare settings, it is important to conduct tailored analyses which reflect the clinical discipline and complexity of the data in the hope of providing clinically meaningful results that identify potential areas for care improvement or resource allocation.

Hip fractures (or fractures involving the proximal femur) have high levels of mortality. In 2007 in England and Wales, 10.9% of patients admitted with hip fracture died within 30-days of admission. The introduction of extensive reforms, audit and a Best Practice Tariff (BPT) saw mortality within these patients fall by 2.5% [14–16]. However, many of the reforms and targets arose from aspirations to provide timely and efficient care rather than on the basis of strong evidence. Despite significant improvements in the care pathway of patients with hip fracture [14], mortality remains high, and the potential to optimise the care pathway is great.

In the context of hip fracture care, there is a paucity of information in relation to the weekend effect. In the available research (see Additional file 1 for systematic search strategy and summary of published literature) there is substantial methodological heterogeneity relating to study size (range 242 to 460,000 participants); temporal definitions of mortality (inpatient, 2/5/30/120 days); definitions of the weekend (e.g. Saturday and Sunday or 4 pm Friday to 4 pm Sunday); exposure of interest (admission, surgery and inpatient stay); settings (national registry, national probability sample, state registry, single centre); healthcare systems (England, Wales, USA, Canada, Germany, Denmark); case-mix adjustment strategies; risk estimates (odds ratios, risk ratios, hazard ratios, not reported or not significant); and publication type (full article, brief correspondence, conference abstract) [2, 3, 5–13, 17–19].

There are no randomised trials available looking at the presence or absence of a weekend effect in hip fracture care. The larger studies (national [3, 5, 7, 10] or state-wide case series [6, 8, 9]) typically used routinely collected data with retrospective disease coding to identify hip fractures and confounders of interest; therefore, heterogeneity in coding practices may result in case misclassification and poor case ascertainment. Smaller studies used prospectively collected data, but were typically underpowered to detect weak effects [11–13, 17–19]. However, the primary limitation common to existing studies is in how the weekend effect is modelled. Nearly all studies attempt to form a binary indicator of weekday versus weekend, thereby assuming any association is collapsible across Saturday and Sunday together or similarly across the weekdays. Of the 14 studies reporting the association between mortality and the day of admission in hip fracture patients, weekend admission was found to increase mortality in one study [13], decrease mortality in one [7], and not be associated in the remaining 12 [2, 3, 5, 6, 8–12, 17–19]. Two studies investigated the association between day of the week of surgery and mortality and found no association [12, 13], whereas only one study investigated the association between the day of death during the inpatient stay and found mortality was lower during the weekend [2].

**Fig. 1:**
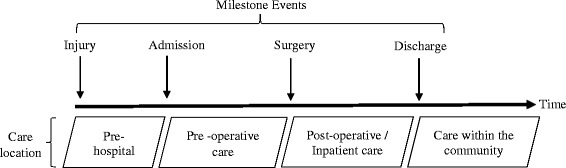
The care pathway of patients admitted for hip fracture

Within hip fracture care, the pathway can be broadly broken into four distinct phases, punctuated by milestones (Fig. 1). The timing of milestones and the care delivered between them may influence a patient’s outcome and therefore require consideration in analyses that investigate mortality following an index event. However, analysis of milestones during the care pathway is complex due to correlations induced by institutional or national targets. For example, one of the requirements of the BPT within England is for a patient to have surgery for hip fracture within 36 hours of admission. Therefore, considering a weekend effect on the basis of admission alone fails to adequately consider the complexity of the patient cohort, care pathway or the correlation between milestones (e.g. timing of admission and surgery).

## Methods

Using Data from the National Hip Fracture Database (NHFD), we investigated the association between the timing of admission, surgery, discharge and mortality at 30-days following the initial admission to hospital for hip fracture in patients admitted to hospital between 2011 and 2014. In addition, we also explored the day of the week of death during the inpatient stay in patients with hip fracture.

### Data source

The NHFD commenced data collection in 2007. Data is estimated to be 95% complete from January 2011 [20]. Patients’ details with traceable NHS number were passed to the NHS Personal Demographics Service, who provided the date of death from the Office for National Statistics.

### Inclusion/exclusion criteria

**Fig. 2:**
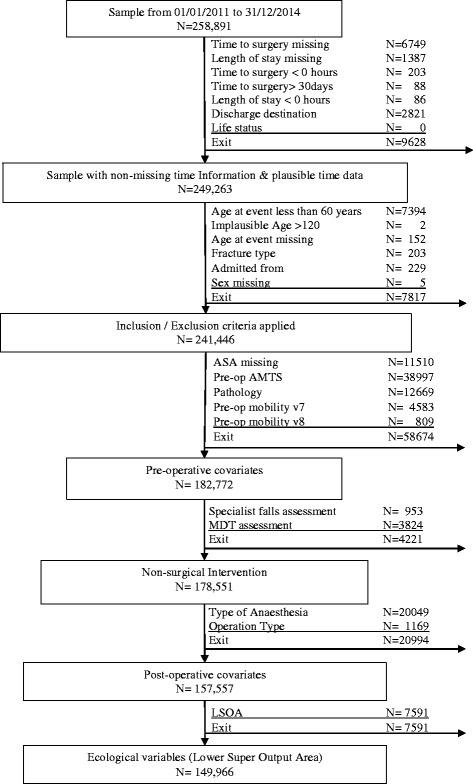
Patient inclusion/exclusions into the study

All individuals admitted with an incident hip fracture between January 1, 2011, and December 31, 2014, and a known date of admission, time of surgery (and surgery within 30 days), and discharge destination were included in the analysis. Patients aged less than 60 and more than 120 years, and with unknown sex were excluded (Fig. 2).

### Primary outcome

The primary outcome is death at 30 days following the initial hospital admission for hip fracture. Death was determined using a combination of Office for National Statistics death records and time of discharge/discharge destination, which also indicates when a patient has died. Contralateral hip fractures in the same patient were considered to be independent events

### Exposures of interest

The primary exposures of interest in this study are the day of the week of admission, surgery, time to surgery, inpatient stay and discharge from the admitted trust. We also investigated whether or not admission, surgery or discharge were within normal working hours (08:00–17:00).

### Confounding factors

Given the well-known seasonal variation in mortality, we adjusted all analyses for the month of admission using dummy indicators and allowed for changes across time using yearly indicators [21]. Pre-existing patient level (age, sex, pre-admission residence, type of fracture, American Society of Anesthesiologists (ASA) grade), non-surgical treatment (falls assessment, multidisciplinary team assessment), surgical (operation type, anaesthetic), discharge destination, and socioeconomic confounding factors were included in the models (see Additional file 1: Table S1 for detailed coding).

### Statistical analysis and sensitivity analyses

Means, standard deviations and interquartile points were used to describe continuous variables. Frequencies and percentages were used to describe categorical variables. The associations between 30-day mortality and time of admission, surgery and discharge were modelled using logistic regression.

Given the large possible number of parameterisations for temporal associations, we initially explored a variety of crude and minimally adjusted models, including a daily effect (dummy indicators for each day of the week); a weekend (Saturday/Sunday) versus weekday effect; an out-of-hour’s effect of admission to hospital or surgery (defining in-hours as 08:00–17:00); and a time from admission to surgery using ordinal, cumulative and log_e_ time parametrisations. We then adopted a pragmatic model building approach carrying forward the most parsimonious variable specifications from the initial analyses and simplifying models where appropriate. In our final models, we replaced binary day-of-week effects with indicator variables representing each day of the week, and performed post-estimation Wald tests comparing if all daily indicators were significantly different from zero. In addition, we also performed post-estimation Wald tests on daily parameter estimates that were significantly different from one another.

Confounding adjustment was conducted incrementally whilst respecting the temporal and causal structure of the care pathway [22]; 11 models were used to explore the associations between admission, surgery and discharge. Model 0 explored the association between the exposures of interest independently of one another and mortality at 30 days. Model 1 explored the association between the exposures of interest independently of one another whilst adjusting for patient-level confounding factors (see above for specification). Model 2 simultaneously explored the exposures of interest whilst adjusting patient-level confounding factors. Model 3 is a parsimonious specification of Model 2. Model 4 is Model 3 adjusted for non-surgical treatment factors. Model 5 is Model 4 adjusted for surgical confounding factors. Model 6 is Model 5 adjusted for socioeconomic position (Additional file 2: Table S1). Given the wide variety of seasonal model specifications, we conducted sensitivity analyses using two alternative seasonal specifications. Model 7 used an elapsed month parameterisation and Model 8 used trigonometric regression (Fourier series) [23, 24].

The association between day of discharge and 30-day mortality was restricted to individuals discharged alive from hospital. Model 9 then investigated day of discharge either via daily indicator variables or as a binary indicator for discharge on a Sunday. Model 10 was further refined by adjusting for discharge destination. Interactions between the day of the week of discharge and discharge destination were explored using likelihood ratio tests.

The incidence of death during the inpatient stay was investigated using Poisson regression. The number of deaths on any given day was derived using the discharge destination. The number of patients in hospital following hip fracture was derived by date of admission and date of discharge and included within the model as an offset parameter. The association between day of the week and death was explored using either daily indicators, or a weekday/weekend specification. We fitted Poisson regression models to daily summary information for all individuals (Model 11), sex-specific daily summaries (Model 12), and age- and sex-specific daily summaries (Model 13). In Models 12 and 13, we performed stratum-specific seasonal adjustments through interactions between month and sex (Model 12) or between month, age and sex category (Model 13). In addition, we explored two methods of modelling seasonality (elapsed month model and a restricted cubic spline approach) [23, 25]. Results are reported as incident rate ratio and 95% confidence intervals. For examples of model specification, see Additional file 1- Modelling Seasonal Specification.

All analyses were conducted in Stata 14.0 (StataCorp LP, College Station, TX).

### Missing data

Despite good data completion rates within the NHFD, item non-response is a problem when adopting complete case analyses. Assuming the data is missing at random, we imputed missing values using multiple imputation with chained equations (MICE). Sex-specific imputation models were derived for each variable that contained missing data. MICE model specification can be found in Additional file 2: Table S2. Ten imputed datasets were generated with a burn-in of 30 repetitions, Monte-Carlo error of parameter estimates of interest, i.e. Sunday surgery, were investigated and were small (Model 5 MICE = 0.0004, with maximal deviation on the odd ratio scale of 0.0027 from the multiple imputation point estimate), and results were combined using mi estimate in Stata.

## Results

Between January 1, 2011, and December 31, 2014, 258,891 patients were admitted to hospitals with a hip fracture. Following the application of inclusion and exclusion criteria, 241,446 cases were available for analysis (Fig. 2).

The average length of stay in hospital in those alive at 30 days was 2.1 days longer in males compared to females (Table 1); 73.1% of hip fractures occurred in females and 30-day mortality was higher in males (10.2% vs. 6.1%; Table 2). The type of fracture, type of residence patients were admitted from, overall condition (ASA grade), pre-operative mobility, pathology, non-surgical treatments received, surgical treatments received, and deprivation were all similar between genders (Table 2 and Additional file 2: Table S3).

**Table 1:** Descriptive statistics of continuous variables

Variable	Sex		N	Mean	(SD)	[25^th^,	50^th^,	75^th^]
Time to surgery (hours)	Male	Alive @ 30 days	58,203	35.1	(36.9)	[18.6,	24.3,	41.3]
Length of stay (days)			58,203	24.0	(23.5)	[10.1,	16.7,	29.8]
Age at event (years)			58,203	81.0	(8.6)	[76.0,	82.0,	87.0]
AMTS pre-op			48,750	7.5	(3.4)	[6.0,	9.0,	10.0]
Time to surgery (hours)		Dead @ 30 days	6608	37.4	(37.4)	[18.9,	25.8,	43.1]
Length of stay (days)			6608	12.2	(8.0)	[5.8,	10.8,	17.4]
Age at event (years)			6608	85.1	(7.6)	[81.0,	86.0,	90.0]
AMTS pre-op			5380	5.7	(3.9)	[1.0,	7.0,	10.0]
Time to surgery (hours)	Female	Alive @ 30 days	165,927	32.0	(31.6)	[18.0,	23.5,	37.5]
Length of stay (days)			165,927	21.9	(116.0)	[9.6,	15.0,	26.0]
Age at event (years)			165,927	83.0	(8.3)	[78.0,	84.0,	89.0]
AMTS pre-op			139,644	7.0	(3.6)	[5.0,	9.0,	10.0]
Time to surgery (hours)		Dead @ 30 days	10,708	34.9	(32.6)	[18.6,	25.0,	41.9]
Length of stay (days)			10,708	11.9	(8.4)	[5.1,	10.2,	17.6]
Age at event (years)			10,708	87.1	(7.5)	[83.0,	88.0,	92.0]
AMTS pre-op			8675	5.2	(3.9)	[1.0,	6.0,	9.0]
Time to surgery (hours)	All	Alive @ 30 days	224,130	32.8	(33.1)	[18.2,	23.8,	38.7]
Length of stay (days)			224,130	22.5	(100.5)	[9.8,	15.3,	27.0]
Age at event (years)			224,130	82.5	(8.4)	[77.0,	84.0,	89.0]
AMTS pre-op			188,394	7.2	(3.6)	[5.0,	9.0,	10.0]
Time to surgery (hours)		Dead @ 30 days	17,316	35.9	(34.5)	[18.7,	25.2,	42.4]
Length of stay (days)			17,316	12.0	(8.3)	[5.3,	10.4,	17.5]
Age at event (years)			17,316	86.3	(7.6)	[82.0,	87.0,	92.0]
AMTS pre-op			14,055	5.4	(3.9)	[1.0,	6.0,	9.0]

*AMTS* Abbreviated Mental Test Score

**Table 2:** Descriptive statistics of categorical variables

Variable	Level	Males	(%)	Females	(%)
Life status at 30 days	Alive/assumed alive	58,203	(89.8)	165,927	(93.9)
	Dead	6608	(10.2)	10,708	(6.1)
	Missing	0	(0.0)	0	(0.0)
Type of fracture	Intracapsular – displaced	32,113	(49.5)	84,782	(48.0)
	Intracapsular – undisplaced	6471	(10.0)	17,597	(10.0)
	Intertrochanteric	21,983	(33.9)	62,478	(35.4)
	Subtrochanteric	3609	(5.6)	10,030	(5.7)
	Other	635	(1.0)	1748	(1.0)
	Missing	0	(0.0)	0	(0.0)
Admitted from	Hospital	3717	(5.7)	6102	(3.5)
	Nursing/Rehab/Residential	11,064	(17.1)	37,397	(21.2)
	Own home/sheltered housing	50,030	(77.2)	133,136	(75.4)
	Missing	0	(0.0)	0	(0.0)
ASA grade	1	1249	(1.9)	3939	(2.2)
	2	14,915	(23.0)	52,145	(29.5)
	3	35,749	(55.2)	93,205	(52.8)
	4	9632	(14.9)	18,445	(10.4)
	5	230	(0.4)	427	(0.2)
	Missing	3036	(4.7)	8474	(4.8)
Pathology	None	59,117	(91.2)	164,707	(93.2)
	Malignancy/Yes	1573	(2.4)	1957	(1.1)
	Atypical	358	(0.6)	1065	(0.6)
	Missing	3763	(5.8)	8906	(5.0)
Pre-operative mobility	Walks without aids	28,042	(43.3)	80,270	(45.4)
	Walks with aids	33,814	(52.2)	89,108	(50.4)
	No functional mobility	1461	(2.3)	3359	(1.9)
	Missing	1494	(2.3)	3898	(2.2)
Multidisciplinary rehabilitation team assessment	No	2690	(4.2)	6537	(3.7)
	Yes	60,341	(93.1)	165,188	(93.5)
	Missing	1780	(2.7)	4910	(2.8)
Specialist fall assessment	Yes	60,012	(92.6)	164,352	(93.0)
	No falls assessment	4274	(6.6)	10,813	(6.1)
	Missing	525	(0.8)	1470	(0.8)
Operation type	Bipolar hemi cemented	6206	(9.6)	15,770	(8.9)
	Bipolar hemi uncemented	1797	(2.8)	4399	(2.5)
	THR cemented	2513	(3.9)	7822	(4.4)
	THR uncemented	793	(1.2)	2180	(1.2)
	Unipolar hemi cemented	16,976	(26.2)	44,773	(25.3)
	Unipolar hemi uncemented	4846	(7.5)	12,470	(7.1)
	Internal fix: cannulated screw/screw	2748	(4.2)	7896	(4.5)
	Internal fix: IM nail	5949	(9.2)	16,043	(9.1)
	Internal fix: sliding hip screw	22,524	(34.8)	64,147	(36.3)
	No operation performed	39	(0.1)	74	(0.0)
	Other	375	(0.6)	963	(0.5)
	Missing	45	(0.1)	98	(0.1)
Type of anaesthesia	Spinal	19,294	(29.8)	51,376	(29.1)
	Spinal + (Epi/NB)	6336	(9.8)	16,095	(9.1)
	GA	14,312	(22.1)	38,885	(22.0)
	GA + (Epi/NB)	15,651	(24.1)	44,567	(25.2)
	Other	929	(1.4)	2714	(1.5)
	Missing	8289	(12.8)	22,998	(13.0)
Discharge destination	Dead	7217	(11.1)	11,477	(6.5)
	Acute hospital	817	(1.3)	1612	(0.9)
	Rehab/Residential/Nursing	25,792	(39.8)	77,846	(44.1)
	Own home	30,985	(47.8)	85,700	(48.5)
	Missing	0	(0.0)	0	(0.0)
Out of hours discharge	In-hours discharge	48,685	(75.1)	136,958	(77.5)
	Out-of-hours discharge	16,126	(24.9)	39,677	(22.5)
	Missing	0	(0.0)	0	(0.0)

*Rehab* Rehabilitation, *IM* Intramedullary, *Epi* Epidural, *NB* Nerve Block, *GA* General Anaesthetic, *THR* Total Hip Replacement, *ASA* American Society of Anesthesiologists Physical Status Classification, *Hemi* Hemiarthroplasty

Exploratory analyses investigating key exposures adjusting for patient-level characteristics indicated strong seasonal variation in 30-day mortality. In the unadjusted analysis, where other factors that influence the risk of 30-day mortality are not considered, the day of admission was associated with higher mortality rates at the weekends as opposed to mid-week, with weekend admission averaging a 5% increase in mortality versus weekdays. Day of the week of surgery illustrated much greater variation in mortality than admission across the week. There was no strong evidence of an association between mortality and out of hour’s admission or surgery. An increase in the time to surgery from admission was associated with higher mortality, with strong evidence of an increased risk of mortality for those receiving surgery after 24 hours (Additional file 2: Table S5 and Table S6).

Following simultaneous adjustment for the exposures of interest (month of admission, day of the week of admission and surgery, out-of-hours surgery, and time to surgery) the association between the day of the week of admission and mortality was attenuated, whilst the association between the day of the week of surgery and mortality persisted, as did the association between the time to surgery from admission (Additional file 2: Table S7).

Grouping days of the week of surgery with similar associations (Wald test that Monday–Saturday are equal to one another *P* = 0.4) demonstrated that Sunday surgery was associated with a 9.4% increase in the odds of 30-day mortality. There was no effect of out-of-hours surgery. Surgery more than 24 hours after admission resulted in a 9.4% increase in mortality (Table 3).

**Table 3:** Model 3 – Multivariate adjusted models, using a simplified day-of-the-week coding, adjusted for all listed variables included, and pre-admission patient characteristics (fracture type, ASA, AMTS, Pathology, Mobility) effects not shown; N (multiple imputation) = 241,446, N (complete cases) = 182,772

		Multiple imputation			Complete cases		
Variable	Level	OR	(95% CI)	*P*	OR	(95% CI)	*P*
Year of admission – 2011	0	1			1		
	1	1.007	(0.962, 1.054)	0.77	1.084	(1.021, 1.150)	0.0083
	2	0.922	(0.881, 0.965)	5.1 × 10^–4^	0.983	(0.928, 1.043)	0.57
	3	0.795	(0.759, 0.833)	0	0.849	(0.800, 0.901)	5.5 × 10^–8^
Month of admission	January	1			1		
	February	0.976	(0.906, 1.053)	0.53	1.001	(0.914, 1.097)	0.98
	March	0.899	(0.834, 0.969)	0.0052	0.923	(0.843, 1.011)	0.085
	April	0.879	(0.816, 0.948)	8.3 × 10^–4^	0.885	(0.807, 0.969)	0.0084
	May	0.797	(0.739, 0.861)	7.0 × 10^–9^	0.820	(0.748, 0.899)	2.5 × 10^–5^
	June	0.758	(0.701, 0.820)	3.9 × 10^–12^	0.796	(0.725, 0.874)	1.7 × 10^–6^
	July	0.753	(0.696, 0.814)	1.3 × 10^–12^	0.746	(0.678, 0.820)	1.2 × 10^–9^
	August	0.792	(0.733, 0.855)	3.2 × 10^–9^	0.820	(0.748, 0.900)	2.7 × 10^–5^
	September	0.844	(0.781, 0.911)	1.4 × 10^–5^	0.889	(0.812, 0.973)	0.011
	October	0.835	(0.774, 0.900)	3.1 × 10^–6^	0.847	(0.774, 0.927)	3.3 × 10^–4^
	November	0.815	(0.755, 0.879)	1.4 × 10^–7^	0.830	(0.758, 0.909)	5.6 × 10^–5^
	December	0.940	(0.875, 1.011)	0.095	0.983	(0.902, 1.071)	0.69
Sunday surgery	MTWTFS surgery	1			1		
	Sunday surgery	1.094	(1.043, 1.148)	2.2 × 10^–4^	1.078	(1.018, 1.141)	0.010
Out-of-hours surgery	In hours	1			1		
	Out of hours	1.010	(0.941, 1.084)	0.79	1.041	(0.956, 1.134)	0.35
Time to surgery ≤ 24 hours	≤24 hours	1			1		
	> 24 hours	1.094	(1.059, 1.130)	6.0 × 10^–8^	1.090	(1.049, 1.133)	9.3 × 10^–6^

*MTWTFS* Monday, Tuesday, Wednesday, Thursday, Friday, Saturday, *OR* Odds Ratio, *CI* Confidence Interval. Omnibus Wald test for year of admission: (CC) *P* < 1.0 × 10^–16^; (MI) *P* < 1.0 × 10^–16^. Omnibus Wald test for month of admission (CC) *P* < 3.4 × 10^–14^; (MI) *P* < 1.0 × 10^–16^

Adjusting models for non-surgical treatments, surgical treatments and socioeconomic position had little effect on the association between Sunday surgery, out-of-hours surgery, surgery more than 24 hours after admission and 30-day mortality (Table 4 and Additional file 2: Table S8). Modelling seasonal changes using either an elapsed month model or trigonometric regression had little effect on the estimated associations of interest (Additional file 2: Table S9).

**Table 4:** Multivariate adjusted models investigating the association between time of surgery and mortality at 30 days. Models adjusted for non-surgical interventions (Model 4) N (multiple imputation) = 241,446, non-surgical interventions and surgical treatments (Model 5) N (multiple imputation) = 241,446, non-surgical interventions, surgical treatments and Index of Multiple Deprivation (Model 6) N = 225,324

		Model 4			Model 5			Model 6		
		OR	(95% CI)	*P*	OR	(95% CI)	*P*	OR	(95% CI)	*P*
Month of year	January	1			1			1		
	February	0.991	(0.918, 1.069)	0.81	0.988	(0.916, 1.066)	0.75	0.987	(0.912, 1.069)	0.75
	March	0.912	(0.846, 0.984)	0.017	0.911	(0.844, 0.982)	0.015	0.923	(0.853, 0.998)	0.044
	April	0.884	(0.819, 0.953)	0.0014	0.880	(0.816, 0.950)	9.9 × 10^–4^	0.900	(0.832, 0.973)	0.0085
	May	0.808	(0.748, 0.872)	5.8 × 10^–8^	0.806	(0.746, 0.871)	4.4 × 10^–8^	0.818	(0.755, 0.886)	8.5 × 10^–7^
	June	0.776	(0.717, 0.839)	2.6 × 10^–10^	0.776	(0.718, 0.840)	2.8 × 10^–10^	0.797	(0.735, 0.864)	4.5 × 10^–8^
	July	0.769	(0.710, 0.832)	6.9 × 10^–11^	0.770	(0.712, 0.833)	9.3 × 10^–11^	0.788	(0.726, 0.855)	1.2 × 10^–8^
	August	0.799	(0.739, 0.864)	1.7 × 10^–8^	0.799	(0.740, 0.864)	1.7 × 10^–8^	0.819	(0.755, 0.887)	1.1 × 10^–6^
	September	0.862	(0.798, 0.931)	1.6 × 10^–4^	0.863	(0.799, 0.933)	1.9 × 10^–4^	0.881	(0.814, 0.955)	0.0019
	October	0.858	(0.795, 0.926)	8.9 × 10^–5^	0.858	(0.795, 0.926)	8.7 × 10^–5^	0.876	(0.809, 0.948)	0.0011
	November	0.843	(0.781, 0.911)	1.4 × 10^–5^	0.843	(0.781, 0.910)	1.3 × 10^–5^	0.859	(0.794, 0.931)	1.9 × 10^–4^
	December	0.967	(0.899, 1.040)	0.36	0.967	(0.899, 1.040)	0.36	0.987	(0.916, 1.065)	0.74
Sunday surgery	MTWTFS surgery	1			1			1		
	Sunday surgery	1.092	(1.040, 1.145)	3.5 × 10^–4^	1.083	(1.032, 1.137)	0.0011	1.087	(1.035, 1.142)	9.3 × 10^–4^
Out-of-hours surgery	In-hours	1			1			1		
	Out-of-hours	1.008	(0.939, 1.082)	0.83	1.011	(0.942, 1.086)	0.76	1.021	(0.950, 1.099)	0.57
Time to surgery ≤ 24 hours	≤ 24 hours	1			1			1		
	>24 hours	1.080	(1.045, 1.116)	4.0 × 10^–6^	1.088	(1.053, 1.125)	4.4 × 10^–7^	1.120	(1.082, 1.158)	5.9 × 10^–11^

Model 3 = Pre-admission characteristics + Non-surgical interventions; Model 4 = Model 3 + Surgical Treatments; Model 5 = Model 4 + Index of Multiple Deprivation

Pre-admission characteristics = Fracture type, ASA, AMTS, Pathology, Mobility; Non-surgical interventions = Falls Assessment, MDT meeting; Surgical interventions = anaesthetic type, operation type; Index of Multiple Deprivation = Index of Multiple Deprivation Older People England, Index of Multiple Deprivation Older People Wales; Omnibus Wald test for: month of admission (Models 4) *P* = 1.0 × 10^–16^; (Models 5) *P* = 1.0 × 10^–16^ (Model 6) *P* = 7.9 × 10^–15^

*MTWTFS* Monday, Tuesday, Wednesday, Thursday, Friday, Saturday; *OR* odds ratio; *CI* Confidence Interval

Analyses exploring the association between discharge characteristics and 30-day mortality showed substantially higher risk of mortality for discharge on a Sunday, although this was relatively rare (N = 4653 (2.5%) of discharges). Out-of-hours discharge was also associated with elevated mortality. These findings were not attenuated after adjusting for discharge destination (Table 5).

**Table 5:** Multivariate adjusted model investigating the association between the day of discharge from hospital and mortality at 30 days. Model 9 is adjusted for patient characteristics, non-surgical treatments, day of surgery and surgical procedure. Model 10 is further adjusted for discharge destination. N (multiple imputation) = 181,568; N (complete cases) = 122,586

			Model 9			Model 10		
	Discharge specification		OR	(95% CI)	*P*	OR	(95% CI)	*P*
Multiple imputation (MI)	Daily indicators	Sunday	1			1		
		Monday	0.601	(0.484, 0.745)	3.6 × 10^–6^	0.625	(0.504, 0.776)	2.0 × 10^–5^
		Tuesday	0.623	(0.504, 0.768)	1.0 × 10^–5^	0.646	(0.523, 0.797)	4.7 × 10^–5^
		Wednesday	0.599	(0.485, 0.740)	2.0 × 10^–6^	0.622	(0.504, 0.769)	1.1 × 10^–5^
		Thursday	0.642	(0.520, 0.791)	3.4 × 10^–5^	0.666	(0.540, 0.822)	1.5 × 10^–4^
		Friday	0.668	(0.543, 0.822)	1.4 × 10^–4^	0.695	(0.564, 0.856)	6.1 × 10^–4^
		Saturday	0.746	(0.591, 0.942)	0.014	0.760	(0.601, 0.960)	0.021
		In hours	1			1		
		Out of hours	1.193	(1.098, 1.296)	3.0 × 10^–5^	1.180	(1.086, 1.282)	9.5 × 10^–5^
	Sunday discharge	MTWTFS	1			1		
		Sunday	1.572	(1.292, 1.913)	6.4 × 10^–6^	1.515	(1.244, 1.844)	3.6 × 10^–5^
		In hours	1			1		
		Out of hours	1.187	(1.093, 1.290)	4.8 × 10^–5^	1.174	(1.081, 1.276)	1.4 × 10^–4^
Complete cases (CC)	Daily indicators	Sunday	1			1		
		Monday	0.599	(0.456, 0.787)	2.3 × 10^–4^	0.622	(0.473, 0.817)	6.5 × 10^–4^
		Tuesday	0.633	(0.485, 0.825)	7.2 × 10^–4^	0.654	(0.501, 0.853)	0.0017
		Wednesday	0.636	(0.487, 0.829)	8.1 × 10^–4^	0.657	(0.504, 0.857)	0.0020
		Thursday	0.645	(0.495, 0.841)	0.0012	0.668	(0.513, 0.871)	0.0029
		Friday	0.707	(0.544, 0.918)	0.0092	0.733	(0.564, 0.952)	0.020
		Saturday	0.785	(0.586, 1.050)	0.10	0.795	(0.594, 1.065)	0.12
		In hours	1			1		
		Out of hours	1.242	(1.122, 1.376)	3.0 × 10^–5^	1.230	(1.111, 1.363)	6.8 × 10^–5^
	Sunday discharge	MTWTFS	1			1		
		Sunday	1.523	(1.188, 1.951)	8.8 × 10^–4^	1.472	(1.149, 1.887)	0.0023
		In hours	1			1		
		Out of hours	1.234	(1.114, 1.366)	5.3 × 10^–5^	1.222	(1.104, 1.353)	1.1 × 10^–4^

*MTWTFS*, Monday, Tuesday, Wednesday, Thursday, Friday, Saturday; *OR* odds ratio; *CI* Confidence Interval

In hours 08:00 to 17:00

Omnibus Wald test for daily discharge specification (Models 9) (CC) *P* = 0.0018 (MI) *P* = 0.00003; (Models 10) (CC) *P* = 0.0053 (MI) *P* = 0.0002. Omnibus Wald test for MTWTFS days of discharge are equal to one another (Model 9) (CC) *P* = 0.07 (MI) *P* = 0.057; (Model 10) (CC) *P* = 0.10 (MI) *P* = 0.09

**Fig. 3:**
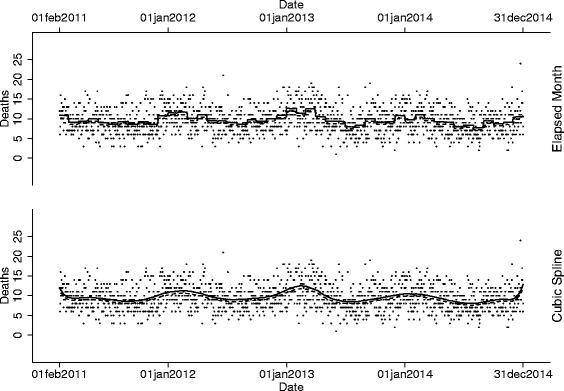
Number of inpatient deaths in patients admitted with hip fracture between February 1, 2011, and December 31, 2014. The upper panel models the incidence of death across the period of interest using an elapsed month model. The lower panel models the incidence of death across the period of interest using cubic splines with knot points every 74 days. Solid lines indicate weekdays, whereas dashed lines indicate the weekend

Mortality during the inpatient stay was investigated between January 1, 2011, and December 31, 2014, in 241,446 hip fractures. The number of hip fractures varies throughout the year; therefore, the number of hip fracture patients in hospital on any given day was calculated and used as the denominator. Due to the elapse between admission and discharge, we utilised data between February 1, 2011, and December 31, 2014, in order to maximise the chance of omitting individuals admitted prior to January 1, 2011 (81% of patients stay less than 31 days in the admitted trust). During the 1430 days of interest, there were 13,461 in-hospital deaths and 4,239,788 bed-days were used within the first 30-days of admission for hip fractures (2965 beds per day). Similar to previous analyses, death was strongly associated with the season, with approximately two more deaths per day in the winter months versus the summer months (Fig. 3). After adjusting for season, the association between mortality and day of the week of inpatient care was investigated. In all models (crude, sex-stratified, sex- and age-stratified) there was strong evidence of fewer deaths at the weekend versus weekdays (–5.5%), with the highest incidence of death occurring on a Wednesday (Table 6).

**Table 6:** The association between day of the week of death and inpatient stay. Models are presented with no adjustment, adjusted for sex, and adjusted for age and sex; 1430 days of interest, 4,239,788 bed days, 13,461 inpatient deaths

		Stratification	1 = No stratification			2 = Stratified by sex			3 = Stratified by age and sex		
Seasonal	Model	Parametrisation	IRR	(95% CI)	*P*	IRR	(95% CI)	*P*	IRR	(95% CI)	*P*
Elapsed month	Day of the week	Sunday	1			1			1		
		Monday	1.033	(0.969, 1.100)	0.322	1.037	(0.974, 1.104)	0.253	1.036	(0.973, 1.104)	0.267
		Tuesday	1.036	(0.972, 1.104)	0.273	1.036	(0.973, 1.102)	0.273	1.035	(0.971, 1.103)	0.289
		Wednesday	1.108	(1.041, 1.180)	0.001	1.111	(1.045, 1.182)	0.001	1.110	(1.043, 1.182)	0.001
		Thursday	1.041	(0.977, 1.109)	0.220	1.043	(0.979, 1.110)	0.192	1.042	(0.978, 1.110)	0.203
		Friday	1.047	(0.983, 1.116)	0.153	1.049	(0.985, 1.116)	0.136	1.048	(0.984, 1.117)	0.144
		Saturday	0.988	(0.927, 1.054)	0.716	0.991	(0.930, 1.056)	0.780	0.991	(0.930, 1.057)	0.791
	Weekend	Weekday	1			1			1		
		Weekend day	0.944	(0.909, 0.980)	0.003	0.944	(0.909, 0.979)	0.002	0.944	(0.909, 0.981)	0.003
Restricted cubic spline	Day of the week	Sunday	1			1			1		
		Monday	1.033	(0.969, 1.100)	0.323	1.037	(0.974, 1.103)	0.257	1.036	(0.973, 1.104)	0.273
		Tuesday	1.034	(0.970, 1.101)	0.306	1.035	(0.972, 1.101)	0.284	1.034	(0.971, 1.101)	0.302
		Wednesday	1.103	(1.037, 1.174)	0.002	1.107	(1.041, 1.177)	0.001	1.106	(1.040, 1.177)	0.001
		Thursday	1.037	(0.974, 1.105)	0.257	1.040	(0.977, 1.107)	0.219	1.039	(0.976, 1.107)	0.231
		Friday	1.044	(0.980, 1.112)	0.187	1.045	(0.982, 1.112)	0.166	1.045	(0.981, 1.113)	0.175
		Saturday	0.986	(0.925, 1.052)	0.673	0.990	(0.929, 1.054)	0.744	0.990	(0.928, 1.055)	0.756
	Weekend	Weekday	1			1			1		
		Weekend day	0.946	(0.911, 0.982)	0.004	0.945	(0.911, 0.981)	0.003	0.946	(0.911, 0.982)	0.004

There was no significant evidence of an interaction between stratification variables and day of the week parameterisation (likelihood ratio test, *P* > 0.1), IRR = Incidence Rate Ratio, CI = Confidence Interval. Omnibus Wald test of day of the week, Elapsed Month seasonal specification: (No stratification) *P* = 0.0128; (Stratified by sex) *P* = 0.0096; (Stratified by age and sex) *P* = 0.0125. Omnibus Wald test of day of the week, Restricted Cubic Spline seasonal specification: (No stratification) *P* = 0.020; (Stratified by sex) *P* = 0.013; (Stratified by age and sex) *P* = 0.017. Omnibus Wald test for MTWTF day of inpatient stay are equal to one another, Elapsed Month seasonal specification (No stratification) *P* = 0.13, (Stratified by sex) *P* = 0.12, (Stratified by age and sex) *P* = 0.13, Omnibus Wald test for MTWTF day of inpatient stay are equal to one another, Restricted Cubic Spline seasonal specification (No stratification) *P* = 0.17, (Stratified by sex) *P* = 0.14, (Stratified by age and sex) *P* = 0.15

## Discussion

In 241,446 hip fractures admitted to hospital between January 1, 2011, and December 31, 2014, we have shown that the day of the week of admission is artefactually associated with 30-day mortality and that the crude association observed in unadjusted data is mediated by the day of the week of surgery. Consistent with previous literature, we show delayed surgery is inadvisable [26–28]. However, we novelly demonstrate that surgery that occurs 24 hours or more after admission to hospital, in a system where 50% of patients receive surgery within 24 hours and 72% within 36 hours, is associated with a 9.4% increase in the odds of 30-day mortality. Similar to previous literature, we find no association between out-of-hours surgery and increases in mortality [29, 30] and, in contrast, we show that Sunday surgery is associated with a 9.4% increase in the odds of 30-day mortality [12, 13]. During the inpatient stay, there is a 5.5% lower incidence of death at the weekend versus weekdays, and the highest incidence of death occurs on a Wednesday. Uniquely, we show there is a 51.5% increase in odds of 30-day mortality for patients discharged on a Sunday, and out of hours discharges are associated with a 17.4% increase in 30-day mortality.

Unlike many other studies based on national data [1–3, 5], the NHFD is a disease-specific prospective national audit and contains detailed information that forms the basis of extensive and relevant risk adjustment models conducted across the care pathway. Furthermore, analyses are not dependent on generic coding practices which indicate specific disease states or co-morbidities; therefore, data acquisition is much more likely to be consistent across the country. Similarly, as the NHFD has many mandatory fields, data completeness is very good for key exposures, outcomes and confounders.

Despite the exceptional size of registry studies and national audits, which typically form the basis of weekend effect research, the ability to make causal inferences from observational epidemiological studies are limited. Whilst we have shown a differential association between Sunday surgery, surgery within 24 hours of admission, the day of discharge and 30-day mortality, it is not clear why these effects occur or how they might be modified. Interpretation of results is complex due to the influence of institutional and national targets. For example, the introduction of a BPT that includes surgery within 36 hours of admission will inevitably induce a correlation between the time of admission and surgery [15]. Therefore, results should be interpreted cautiously and only whilst considering the influence of other critical milestones in the care pathway, including, for example, injury, admission, surgery and discharge from hospital. The care standards driven by the BPT in England and Wales may make the findings of this study less generalisable to other healthcare systems. Finally, despite our attempts to perform extensive case mix adjustment and sensitivity analyses, we cannot be sure that the associations we observe do not depend on unmeasured confounding factors.

Despite popular belief of a generalised weekend effect [1–3, 5] we have shown, using a disease-specific register, that care is not universally inferior at the weekend. We illustrate how simple analyses focusing solely on the day of admission may be confounded by other important milestones in a patient’s care pathway. Therefore, when referring to a weekend effect, it is critical that inferences are made to a specified event or interval within the care pathway.

In the context of hip fractures, it is not clear what the causal mechanisms are which underpin the observed associations seen with delayed surgery, Sunday surgery, Sunday discharges or out-of-hours discharges. There may be an association with the resources available at different times, for example, the provision of orthogeriatrician-led post-hip fracture care has been shown to reduce mortality and this service is not universally available at the weekend [31]. However, in the short/medium term following surgery, the risk of death at the weekend is lower compared to that of weekdays, which suggests care on the ward is likely to be at least equivalent to the care received during the week.

We have shown an exceptionally large increase in the odds of death following discharge on a Sunday and out of hours discharges. Discharge on a Sunday is unusual; therefore, the rationale of such practice is not clear. However, this highlights the importance of effective transitions between acute care providers and care within the community. Whilst the causal mechanisms underpinning these associations are not clear, the large increase in odds of death suggests that these practices should be avoided, and that research into the transition between acute hospitals and the community may prove effective in reducing mortality.

Despite the observed differences in mortality across the care pathway and the suggestion of modifiable risk factors, it is not clear whether any interventions will be effective in reducing absolute mortality in a system with finite financial and human resources in the short or medium term.

## Conclusion

The evidence for a generalised weekend effect in patients with hip fracture is not compelling. We observed an increase in the risk of 30-day mortality for those receiving surgery or being discharged on a Sunday. However, the incidence of death at the weekend is lower than that of weekdays, suggesting it is at least equivalent to care delivered on weekdays. Furthermore, we demonstrate that surgery within 24 hours following admission is associated with a reduction in mortality.

In a healthcare system with finite financial and personnel resources, it is unwise to universally redistribute resources across the week without first considering the differences in the current provision of care. The lack of a generalised Saturday and Sunday effect suggests that resource distribution on a Sunday with reference to operations that occur on a Sunday and discharges is somehow different from a Saturday. Reasons for those differences should be investigated and minimised to reduce mortality. This research highlights the importance of the transition of care between acute hospitals and the community, and the necessity of community care providers and acute hospitals to coordinate a smooth transition into the community.

Finally, redistribution of resources will ultimately mean the removal of resource in one area and reallocation to another; therefore, the net effect on any outcome of interest is unclear. Careful monitoring of new interventions is required to ensure any changes result in a net reduction in mortality and are cost-effective and safe.

## Additional files

Additional file 1: Summary information on modelling strategy, Multiple Imputation via Chained Equations specification, additional Descriptive statistics, and sensitivity analyses. (DOCX 60 kb)

Additional file 2: Sytematic Search Strategy used in literature review and summary results of systematic review. Description of seasonal modelling approach. (DOCX 95 kb)

## Abbreviations

95% CI: 95% confidence interval
ASA: American Society of Anesthesiologists
MICE: Multiple imputation with chained equations
NHFD: National Hip Fracture Database of England and Wales
OR: Odds Ratio
