# Supplementary material for: The association between the day of the week of milestones in the care pathway of patients with hip fracture and 30-day mortality: findings from a prospective national registry – The National Hip Fracture Database of England and Wales
Source: BMC Med. 2017 Mar 27;15:62. doi: 10.1186/s12916-017-0825-5 (PMC5367007; doi:10.1186/s12916-017-0825-5)
Supplement: Supplementary file 1 — Summary information on modelling strategy, Multiple Imputation via Chained Equations specification, additional Descriptive statistics, and sensitivity analyses. (DOCX 60 kb) [file 12916_2017_825_MOESM1_ESM.docx]

**Supplementary Material: Systematic Search and Summary Information**

**Structured Search Strategy implemented in Medline and Embase using Ovid Silver Platter**

Hip Fracture

( (femoral.mp OR femur.mp OR hip.mp OR osteoporotic.mp or bone.mp or Subcapital.mp or inter?trochanteric.mp or sub?trochanteric.mp or intra?capsular.mp or extra?capsular.mp or per?trochanteric.mp ) AND fracture$.mp) OR femoral neck fractures/ OR osteoporotic fractures/ OR hip fractures/ OR femoral fractures/ OR fractures, bone/

AND

Day of the week

(Day.mp AND Week.mp) OR (Time.mp AND Day.mp) OR Weekend.mp OR Weekday.mp OR Time factors/ OR earl* surgery.mp OR delay* surgery.mp

AND

Care Pathway

Patient Admission/ OR Patient Discharge/ OR Length of stay/ OR Perioperative Care/ OR

Admission.mp OR Discharge.mp OR Surgery.mp OR Operation.mp OR

inpatient stay.mp OR hospital stay.mp

AND

Mortality

Hospital Mortality/ OR Survival Rate/ OR Death/ OR Mortality/ OR Death.mp OR

Mortality.mp

*keyword*.mp indicates multi post fields including (Title, Original Title, Abstract, Subject Heading, Name of Substance and Registry Word fields.

*Keyword*/ indicates a Medical Subject Heading (MeSH) search term

Date Range: Inception to 1^st^ March 2016

Search Results: 806 Abstracts Retrieved

Supplementary Material 1.Summary Characteristics of articles reporting associations between day of the week effects and mortality.

| Pub  Type | Publ.  Date | First  Author | Time Frame | Country | Setting | Mortality  Definition | Weekend Definition | Exposure | N | Crude mortality | Risk adjustment | Adjusted Effect |
| --- | --- | --- | --- | --- | --- | --- | --- | --- | --- | --- | --- | --- |
| JA | 2001 | Bell^1^ | 01/04/1988 –  31/03/1997 | Canada | State  wide | In hospital | S&S | Admis | 59,670 | 7% weekday  6% weekend | Age, Sex, Charlson | OR: 0·97  [95%CI:0·90, 1·04]) |
| JA | 2004 | Cram^2^ | 01/01/1998-31/12/1998 | USA | California state | In hospital | S&S | Admis | 22,001 | NR | Age, sex, and race and comorbidity | OR: 1·13 [95%CI:0·97, 1·32] |
| JA | 2006 | Foss^3^ | 09/2002 –  07/2004 | Denmark | Single  centre | 5 day OR  30 days | 4pm Friday  to  4pm Sunday | Admis | 600 | 2% wkday @ 5 days  3% wkend @ 5 days  11% wkday @ 30 days  13% wkend @ 30 days | Not described | NRNS |
| JA | 2010 | Aylin^4^ | April 2005  and 31 March 2006 | UK | NHS acute hospitals | In hospital death at any time during stay. | S&S | Admis | 53,153 | 13·4% weekday  12·9% weekend | Adjusted for age, sex, deprivation quintile and comorbidity. | OR: 0·98 [95%CI:0·92, 1·04]  p value 0·490 |
| JA | 2010 | Clarke^5^ | 2002/2003 –  2006/2007 | Australia | State  wide | 2 day OR  30 days | S&S | Admis | 4,183 | 1·1% wkday @ 2 days  1·3% wkend @ 2 days  6% wkday @ 30 days  4·9% wkend @ 30 days | Age, sex, indigenous, SES, remoteness, co-morbidities | RR: 1·21  [ 95% CI:0·46,1·93] @ 2 days  RR: 0·78  [ 95% CI:0·54,1·03] @ 30 days |
| JA | 2012 | Freemantle^6^ | April 2009 – March 2010 | UK | All NHS hospitals | 30 day mortality as an in or out patient | All Days (compared with Wednesday) | Admission &  Hospital Stay | Reason for admission not recorded  4,253 deaths. | NR for hip fractures | Age, sex, ethnicity, emergency / planned admission, source of admission (e.g. home or inter-hospital transfer, diagnostic group, number of previous emergency admissions;  number of previous complex admissions; Charlson Index of co-morbidities; social deprivation;  hospital trust; day of the year (seasonality). | Hazard Ratio (Admission):  Sun·1·07 [95%CI:0·95, 1·19]  Mon·0·98 [95%CI: 0·87, 1·09]  Tue·0·99 [95%CI:0·89,1·11]  Wed· [REFERENCE]  Thu·0·97 [95%CI:0·87,1·09]  Fir·1·04 [95%CI:0·93,1·16]  Sat·0·94 [95%CI:0·94,1·05]  Hazard Ratio (Hospital Stay):  Sun·0·82 [95%CI:0·73, 0·91]  Mon·0·91 [95%CI: 0·82, 1·02]  Tue·0·89 [95%CI:0·79, 0·99]  Wed· [REFERENCE]  Thu·0·85 [95%CI:0·76, 0·95]  Fir·0·87 [95%CI:0·78, 0·97]  Sat·0·88 [95%CI:0·79, 0·98] |
| CA | 2012 | Akhtar^7^ | 2009-2012? | England | Single  centre | ? | NR | Admis | 1,039 | 7% wkday  5·2% wkend | NR | OR 0·722 ?[95%CI:0·39,1·33 ]?  NS |
| JA | 2012 | Daugaard^8^ | 2003-2010 | Denmark | National | In hospital  30 Day |  | Admis. | 38,020 | NR | Not Described | NRNS |
| CA | 2013 | Mathews^9^ | 01/04/2009 –  31/09/2011 | England | Single  centre | 30 days  120 days | NR | Admis. | 816 | NR | NR | NS @ 30days (p=0·842) or 12 days (p=0·425) |
| CA | 2014 | Monem^10^ | 2009-2012  2012-2013 | England | Single  centre | 30 day | NR | Admis. | 1,489 | 6·54% overall (09/12)  4·9% overall (12/13) | NR | OR: 0·72  [95% CI:0·39, 1·32] @ (09/12)  OR: 1·58  [95% CI:0·62, 3·99] @ (12/13) |
| JA | 2014 | Thomas^11^ | 07/2009-  02/2013 | England | Single  centre | 30 day | S&S | Admis. &  Surgery | 2,987 | 8·7% overall | Age, Gender, ASA, TTS | OR 1·4  [95% CI:1·02, 1·9] (admission)  OR: 1·2  [95% CI:0·8, 1·7] (surgery) |
| JA | 2015 | Boylan^12^ | 1998-2010 | USA | 20% National Sample | In hospital | NR | Admis. | 344,989 | NR | Age, sex, race, insurance, N comorbidities, hospital region, fracture location, hospital size, hospital size, teaching status | OR 0·94  [95%CI: 0·89,0·99] |
| JA | 2015 | Muhm^13^ | 01/01/2012-  31/12/201 | Germany | Single Centre | Clinical mortality | NR | Admis. &  Surgery | 242 | 8·3% | NR | NRNS |
| BC | 2015 | Roberts^14^ | 2004-2012 | England  Wales | National | 30 day | NR | Admis | 461,790 | 7·98% | Age, sex, co-morbidities | OR 1·019  [95%CI:0·994, 1·044] England  OR 1·086  [95%CI:0·983, 1·200] Wales |

JA= Journal article, CA=Conference Abstract, BC=Brief correspondence, OR= Odds Ratio, RR=Risk Ratio, NR=Not reported, NR&NS= Not reported and not significant, wkend=weekend, wkday=weekday, S&S=Saturday and Sunday, Admis.=Admission.

**Modelling Seasonal Specification**

Elapsed Month Model

An elapsed month model specification, models seasonal variation by including dummy variables for each elapsed month in the period of interest.^15^ A Poisson model with an offset is described below.

$$ln\left( D_{i} \right)=\hat{\beta_{0}}+\hat{\beta_{1}}Weekend+\hat{\beta_{2}}Mar2011+\hat{\beta_{3}}April2011+\ldots+\hat{\beta_{49}}Dec2014+log(n_{i})$$

$D_{i}$ indicates the number of expected deaths on the ith day, $\hat{\beta_{0}}$ is an estimate of the intercept,$\hat{\beta_{1}}$ is the parameter estimate for the log-incidence rate ratio between death and weekend days. $\hat{\beta_{2}}$ to $\hat{\beta_{49}}$ represent the log-incidence rate ratio in monthly varition in mortality between the 1^st^ March 2011 and 31^st^ December 2014 compared to the reference month 1^st^ February 2011.

Trigonometric Regrssion

Trigonometric regression (Fourier series) uses a series of sine and cosine pairs to model season variation ^16^ across the period of interest. A Poisson model with an offset is described below.

$$ln\left( D_{i} \right)=\hat{\beta_{0}}+\hat{\beta_{1}}Weekend+\sum_{j=1}^{j} \hat{s_{j}}sin\left( 2j\pi t_{i} \right)+\sum_{j=1}^{j} \hat{c_{j}}cos\left( 2j\pi t_{i} \right)+log(n_{i})$$

$D_{i}$ indicates the number of expected deaths on the ith day, $\hat{\beta_{0}}$ an estimate of the intercept.$\hat{\beta_{1}}$ is the parameter estimate for the log-incidence rate ratio between death and weekend days. $\hat{s_{j}}$ and $\hat{c_{j}}$ are coefficients estimated from the data. $t_{i}$ is time measured as a fraction of a year, and $\pi$ is 3.14, *j* indexes the *jth* pair of sine and cosine function, and the offset ($n_{i})$ is the number of individuals at risk on the *ith* day of the period of interest.

Cubic Spline Approach

Cubic splines were generated using the user written stata package flexcurv.^17^ flexcurv uses scale invariant bsplines with reference points. Knot reference points (*j)* were placed every 74 days (*t*) from the 1^st^ February 2011, to 31^st^ December 2014.$D_{i}$ indicates the number of expected deaths on the ith day.$\hat{\beta_{1}}$ is the parameter estimate for the association between death and weekend days. $\hat{\alpha_{j}}$ is the estimated coefficient which represents the ln incidence rate of deaths for those at risk on the day of the knot point assuming it is a weekday. Similarly, $\hat{\beta_{1}}$ is the estimated ln incidence rate ratio of deaths on weekend days vs weekdays.$P_{k}$ represents the order of the spline i.e k= 1 (linear), 2 (quadratic), 3 (cubic), and $t;b_{j}$ reprents the spline interval, full details of spline generation is well document by Newson^17^. A Poisson model with an offset is described below.

$$ln\left( D_{i} \right)=\hat{\beta_{1}}Weekend+\sum_{j=1}^{J} \hat{\alpha_{j}}P_{k} \left( t;b_{j} \right)+log(n_{i})$$

**References**

1. Bell CM, Redelmeier DA. Mortality among patients admitted to hospitals on weekends as compared with weekdays. *N Engl J Med* 2001; **345**(9): 663-8.

2. Cram P, Hillis SL, Barnett M, Rosenthal GE. Effects of weekend admission and hospital teaching status on in-hospital mortality. *Am J Med* 2004; **117**(3): 151-7.

3. Foss NB, Kehlet H. Short-term mortality in hip fracture patients admitted during weekends and holidays. *Br J Anaesth* 2006; **96**(4): 450-4.

4. Aylin P, Yunus A, Bottle A, Majeed A, Bell D. Weekend mortality for emergency admissions. A large, multicentre study. *Qual Saf Health Care* 2010; **19**(3): 213-7.

5. Clarke MS, Wills RA, Bowman RV, et al. Exploratory study of the 'weekend effect' for acute medical admissions to public hospitals in Queensland, Australia. *Intern Med J* 2010; **40**(11): 777-83.

6. Freemantle N, Richardson M, Wood J, et al. Weekend hospitalization and additional risk of death: an analysis of inpatient data. *J R Soc Med* 2012; **105**(2): 74-84.

7. Akhtar Z, Lisk R. Hip fracture patients admitted over the weekend - Do they do worse in our hospital? *Eur Geriatr Med* 2012; **Conference**: 8th Congress of the European Union Geriatric Medicine Society Brussels Belgium. Conference Start: 20120926 Conference End: 8. Conference Publication: (var.pagings). 3 (pp S90).

8. Daugaard CL, Jorgensen HL, Riis T, Lauritzen JB, Duus BR, van der Mark S. Is mortality after hip fracture associated with surgical delay or admission during weekends and public holidays? A retrospective study of 38,020 patients. *Acta Orthop* 2012; **83**(6): 609-13.

9. Mathews JA, Hall J, Harvey R, Vindlacheruvu M, Khanduja V. The effect of timing of admission on time-to-surgery and mortality: Is there a 'weekend effect' in patients admitted to a UK teaching hospital following acute hip fracture. *Eur Surg Res* 2013; **Conference**: 2012 European Society for Surgical Research, ESSR Congress Lille France. Conference Start: 20120606 Conference End: 9. Conference Publication: (var.pagings). 50 (2 SUPPL. 4) (pp 171).

10. Monem M, Iskandarani K, Yeong K, Lisk R. Does the day of being admitted matter in hip fractures? how to address your mortality. *Osteoporos Int* 2014; **Conference**: Osteoporosis Conference 2014 Birmingham United Kingdom. Conference Start: 20141130 Conference End: 202. Conference Publication: (var.pagings). 25 (6 SUPPL. 1) (pp S673).

11. Thomas CJ, Smith RP, Uzoigwe CE, Braybrooke JR. The weekend effect: short-term mortality following admission with a hip fracture. *Bone Joint J* 2014; **96-B**(3): 373-8.

12. Boylan MR, Rosenbaum J, Adler A, Naziri Q, Paulino CB. Hip Fracture and the Weekend Effect: Does Weekend Admission Affect Patient Outcomes? *Am J Orthop* 2015; **44**(10): 458-64.

13. Muhm M, Walendowski M, Danko T, Weiss C, Ruffing T, Winkler H. [Factors influencing course of hospitalization in patients with hip fractures: Complications, length of stay and hospital mortality]. *Z Gerontol Geriatr* 2015; **48**(4): 339-45.

14. Roberts SE, Thorne K, Akbari A, Samuel DG, Williams JG. Weekend emergency admissions and mortality in England and Wales. *The Lancet* 2015; **385**(9980).

15. Bhaskaran K, Gasparrini A, Hajat S, Smeeth L, Armstrong B. Time series regression studies in environmental epidemiology. *Int J Epidemiol* 2013; **42**(4): 1187-95.

16. Cox NJ. Speaking Stata: In praise of trigonometric predictors. *Stata J* 2006; **6**(4): 561-79.

17. Newson RB. Sensible parameters for univariate and multivariate splines. *Stata J* 2012; **12**(3): 479-504.
