# Supplementary material for: The association between the day of the week of milestones in the care pathway of patients with hip fracture and 30-day mortality: findings from a prospective national registry – The National Hip Fracture Database of England and Wales
Source: BMC Med. 2017 Mar 27;15:62. doi: 10.1186/s12916-017-0825-5 (PMC5367007; doi:10.1186/s12916-017-0825-5)
Supplement: Supplementary file 2 — Sytematic Search Strategy used in literature review and summary results of systematic review. Description of seasonal modelling approach. (DOCX 95 kb) [file 12916_2017_825_MOESM2_ESM.docx]

Table S1: Model specification detailing exposure and confounding variables used within the analysis.

| Model | Exposures | Confounding variables |
| --- | --- | --- |
| 0 | ⊥ (Year of admission, month of admission,  DOW of admission,  weekend admission,  out of hours admission,  DOW of surgery,  weekend surgery,  out of hours surgery,  TTS including the following parameterisation, identity, log_e_, ordered[ 24/36/48/60/72], categorical[24/36/48/60/72], binary[>24/>36/>48/>60/>72]) |  |
| 1 | ⊥ (Year of admission, month of admission,  DOW of admission,  weekend admission,  out of hours admission,  DOW of surgery,  weekend surgery,  out of hours surgery,  TTS including the following parameterisation, identity, log_e_, ordered[ 24/36/48/60/72], categorical[24/36/48/60/72], binary[>24/>36/>48/>60/>72]) | Patient Level confounding factors |
| 2 | Year of admission, month of admission  DOW of admission, DOW of surgery, out of hours surgery, TTS binary[>24] | Patient Level confounding factors |
| 3 | Year of admission, month of admission  Sunday surgery, out of hours surgery, TTS binary[>24] | Patient Level confounding factors |
| 4 | Model-3 | Patient Level confounding factors, Non-surgical treatment factors |
| 5 | Model-3 | Patient Level confounding factors, Non-surgical treatment factors, Surgical treatment factors |
| 6 | Model-3 | Patient Level confounding factors, Non-surgical treatment factors, Surgical treatment factors,  SEP |
| 7 | Elapsed month seasonal specification  Sunday surgery, out of hours surgery, TTS binary[>24] | Patient Level confounding factors, Non-surgical treatment factors, Surgical treatment factors |
| 8 | Fourier series seasonal specification  Sunday surgery, out of hours surgery, TTS binary[>24] | Patient Level confounding factors, Non-surgical treatment factors, Surgical treatment factors |
| 9 | Model-3 – Restricted to patients discharged alive from hospital within 30-days | Patient Level confounding factors, Non-surgical treatment factors, Surgical treatment factors |
| 10 | Model-3 + day of the week of discharge– Restricted to patients discharged alive from hospital within 30-days | Patient Level confounding factors, Non-surgical treatment factors, Surgical treatment factors  Discharge destination |

⊥-Independently, DOW- Day of the week, TTS- time to surgery , Out of hours-<08:00 & >17:00

Patient level confounding factors (Age [60 to 120], sex[male or female], pre-admission residence [own home, hospital, Nursing/rehabilitation/residential home], type of fracture [Intracapsular undisplaced, Intracapsular displaced, Intertrochanteric, subtrochanteric, other], ASA grade[I+II, III, IV+V], Pre-operative AMTS score, Pathology[None, Malignancy, atypical], Pre-operative mobility [walks without aid, walks with aid, no functional mobility]).

Non-surgical treatment factors( Falls assessment [yes/no], Multi-Disciplinary Team Assessment [yes/no]).

Surgical treatment factors(Anaesthetics [Spinal, Spinal and epidural or nerve block, general anaesthetic, general anaesthetic + epidural or nerve block], Operation type [Unipolar un/cemented, Bipolar un/cemented, total hip replacement un/cemented, Internal fixation cannulated screw, inter medullary nail, sliding hip screw, no operation]).

Social economic position (Deciles of Income Deprivation Affecting Older People Index, Welsh Index Multiple Deprivation).

Discharge confounding factors (discharge destination [own home, acute hospital, rehab/residential/nursing home]).

Table S2: Multiple Imputation via Chained Equations Model (MICE) model specification, detailing the outcome (variable with missing data), the link function, and the predictor variables. Ten sex specific imputed data sets were generated with a burnin of 30 repetitions.

| Outcome | Link function | Predictors | | | | | | | | | |
| --- | --- | --- | --- | --- | --- | --- | --- | --- | --- | --- | --- |
|  |  | i.Death | c.Age at event | c.Pre-op AMTS | i.Admitted From | i.Pre-op mobility | i.Fracture type | i.Pathology | i.ASA Grade | i.Operation | i.Dow of admission |
| Pre-op AMTS | linear | ✓ | ✓ |  |  | ✓ |  | ✓ | ✓ |  |  |
| Admitted From | mlogit | ✓ | ✓ | ✓ |  | ✓ |  | ✓ | ✓ |  |  |
| Pre-op mobility | ologit | ✓ | ✓ | ✓ | ✓ |  |  | ✓ | ✓ |  |  |
| Fracture type | mlogit | ✓ | ✓ | ✓ | ✓ | ✓ |  | ✓ | ✓ | ✓ |  |
| Pathology | mlogit | ✓ | ✓ | ✓ | ✓ | ✓ |  |  | ✓ |  |  |
| ASA Grade | ologit | ✓ | ✓ | ✓ | ✓ | ✓ |  | ✓ |  |  |  |
| Falls Assessment | logit | ✓ | ✓ | ✓ |  | ✓ |  |  | ✓ | ✓ | ✓ |
| MDT | logit | ✓ | ✓ |  |  | ✓ |  |  | ✓ | ✓ | ✓ |
| Anaesthetic | mlogit | ✓ | ✓ | ✓ |  |  | ✓ |  | ✓ | ✓ |  |
| Operation | mlogit | ✓ | ✓ |  |  |  | ✓ |  | ✓ |  |  |

AMTS-Abbreviated Mental Test Score, DOW-Day of the week, MDT-Multi-disciplinary Team, i.- indicator variable parameterisation, c.-continuous parameterisation

Table S3: Descriptive Statistics of Time to surgery from admission by day of the week

|  |  | Variable | N | Mean | SD | [25^th^ , | 50^th^, | 75^th^] |
| --- | --- | --- | --- | --- | --- | --- | --- | --- |
| Male | Alive @30-days | Sunday | 7083 | 32.2 | (27.8) | [18.3, | 24.4, | 40.5] |
|  |  | Monday | 8495 | 37.8 | (38.0) | [18.8, | 26.1, | 45.3] |
|  |  | Tuesday | 8909 | 35.1 | (38.8) | [18.5, | 23.9, | 39.3] |
|  |  | Wednesday | 8758 | 35.7 | (39.6) | [18.9, | 24.4, | 40.8] |
|  |  | Thursday | 8539 | 35.8 | (40.6) | [18.7, | 24.3, | 41.2] |
|  |  | Friday | 8596 | 36.3 | (39.3) | [18.9, | 24.4, | 41.8] |
|  |  | Saturday | 7823 | 31.8 | (29.5) | [18.1, | 23.5, | 38.7] |
|  | Dead @30-days | Sunday | 825 | 32.0 | (22.3) | [18.3, | 25.4, | 41.5] |
|  |  | Monday | 1008 | 40.3 | (42.3) | [19.0, | 26.3, | 45.4] |
|  |  | Tuesday | 974 | 37.4 | (36.2) | [18.6, | 25.9, | 42.4] |
|  |  | Wednesday | 959 | 39.8 | (40.2) | [19.3, | 26.3, | 43.7] |
|  |  | Thursday | 992 | 40.5 | (44.4) | [19.5, | 26.3, | 45.7] |
|  |  | Friday | 963 | 36.7 | (38.2) | [18.3, | 24.9, | 43.1] |
|  |  | Saturday | 887 | 34.2 | (29.8) | [18.8, | 24.9, | 41.3] |
| Female | Alive @30-days | Sunday | 20973 | 30.3 | (26.0) | [17.9, | 23.4, | 38.1] |
|  |  | Monday | 24424 | 34.0 | (32.5) | [18.2, | 24.5, | 41.8] |
|  |  | Tuesday | 24617 | 32.5 | (32.5) | [18.0, | 23.3, | 35.5] |
|  |  | Wednesday | 24322 | 32.5 | (33.9) | [18.2, | 23.6, | 36.0] |
|  |  | Thursday | 24398 | 32.3 | (34.4) | [18.1, | 23.5, | 36.4] |
|  |  | Friday | 24721 | 32.1 | (32.3) | [18.1, | 23.6, | 37.0] |
|  |  | Saturday | 22472 | 30.0 | (26.9) | [17.9, | 23.1, | 35.3] |
|  | Dead @30-days | Sunday | 1432 | 33.1 | (29.9) | [18.7, | 25.3, | 40.9] |
|  |  | Monday | 1554 | 36.8 | (32.4) | [18.7, | 26.4, | 45.7] |
|  |  | Tuesday | 1618 | 34.9 | (32.2) | [18.6, | 24.8, | 41.2] |
|  |  | Wednesday | 1555 | 35.4 | (34.2) | [18.8, | 25.1, | 41.7] |
|  |  | Thursday | 1576 | 36.4 | (38.7) | [18.7, | 25.0, | 41.8] |
|  |  | Friday | 1550 | 35.5 | (33.8) | [18.0, | 24.3, | 41.8] |
|  |  | Saturday | 1423 | 31.7 | (24.4) | [18.4, | 24.5, | 39.1] |
| All | Alive @30-days | Sunday | 28056 | 30.8 | (26.5) | [18.0, | 23.7, | 38.8] |
|  |  | Monday | 32919 | 35.0 | (34.0) | [18.4, | 24.9, | 42.7] |
|  |  | Tuesday | 33526 | 33.2 | (34.3) | [18.1, | 23.4, | 36.3] |
|  |  | Wednesday | 33080 | 33.3 | (35.5) | [18.4, | 23.8, | 37.5] |
|  |  | Thursday | 32937 | 33.2 | (36.1) | [18.3, | 23.7, | 38.0] |
|  |  | Friday | 33317 | 33.2 | (34.3) | [18.3, | 23.8, | 38.5] |
|  |  | Saturday | 30295 | 30.5 | (27.6) | [17.9, | 23.2, | 36.0] |
|  | Dead @30-days | Sunday | 2257 | 32.7 | (27.4) | [18.6, | 25.4, | 41.2] |
|  |  | Monday | 2562 | 38.2 | (36.6) | [18.8, | 26.4, | 45.6] |
|  |  | Tuesday | 2592 | 35.8 | (33.8) | [18.6, | 25.1, | 41.7] |
|  |  | Wednesday | 2514 | 37.1 | (36.7) | [19.0, | 25.5, | 42.2] |
|  |  | Thursday | 2568 | 38.0 | (41.0) | [19.0, | 25.4, | 43.4] |
|  |  | Friday | 2513 | 35.9 | (35.5) | [18.1, | 24.6, | 42.3] |
|  |  | Saturday | 2310 | 32.7 | (26.6) | [18.6, | 24.6, | 40.3] |

Table S4: Distribution of Income Deprivation Affecting Older People Index (2015)* & Welsh Index of Multiple Deprivation (2011).

| Variable | Level | Males | (%) | Females | (%) |
| --- | --- | --- | --- | --- | --- |
| IDAOPI (England) | 1 | 4571 | (8.0) | 10961 | (7.0) |
|  | 2 | 5322 | (9.3) | 14021 | (9.0) |
|  | 3 | 5509 | (9.6) | 15615 | (10.0) |
|  | 4 | 5790 | (10.1) | 16352 | (10.5) |
|  | 5 | 5994 | (10.5) | 16825 | (10.8) |
|  | 6 | 6075 | (10.6) | 17283 | (11.1) |
|  | 7 | 6064 | (10.6) | 17130 | (11.0) |
|  | 8 | 6055 | (10.6) | 16400 | (10.5) |
|  | 9 | 5865 | (10.3) | 15959 | (10.3) |
|  | 10 | 5874 | (10.3) | 15111 | (9.7) |
|  | Missing | 4267 |  | 11871 |  |
| WIMD  (Wales) | 1 | 308 | (8.8) | 810 | (8.7) |
|  | 2 | 378 | (10.8) | 877 | (9.4) |
|  | 3 | 333 | (9.5) | 927 | (10.0) |
|  | 4 | 347 | (9.9) | 1019 | (11.0) |
|  | 5 | 372 | (10.6) | 927 | (10.0) |
|  | 6 | 372 | (10.6) | 973 | (10.5) |
|  | 7 | 358 | (10.2) | 914 | (9.8) |
|  | 8 | 325 | (9.3) | 968 | (10.4) |
|  | 9 | 390 | (11.1) | 1034 | (11.1) |
|  | 10 | 316 | (9.0) | 849 | (9.1) |
|  | Missing | 4267 |  | 11871 |  |

* Due to changes in Local Super Output Areas (LSOA) between 2011 and 2014 in England the average rank of suspended LSOA was imputed using the average rank of surroundings LSOA in the same local authority districts. IDAOPI Income Deprivation Affecting Older People Index; WIMD Welsh Index of Multiple Deprivation.

Table S5: Model-0, Crude association between exposures and 30 day mortality after admission for hip fracture ; N(Multiple Imputation)=241446, N(Complete Cases)=182772 .

|  |  | Multiple Imputation | | | | Complete Cases | | | |
| --- | --- | --- | --- | --- | --- | --- | --- | --- | --- |
| Variable | Level | OR | 95% CI | | p= | OR | 95% CI | | p= |
| Year of admission -2011 | 0 | 1 |  |  |  | 1 |  |  |  |
|  | 1 | 1.035 | (0.991, | 1.081) | 0.12 | 1.124 | (1.061, | 1.191) | 6.7e-05 |
|  | 2 | 0.977 | (0.935, | 1.021) | 0.30 | 1.059 | (1.001, | 1.121) | 0.046 |
|  | 3 | 0.882 | (0.843, | 0.922) | 3.4e-08 | 0.963 | (0.910, | 1.019) | 0.19 |
| Month of admission | January | 1 |  |  |  | 1 |  |  |  |
|  | February | 0.971 | (0.903, | 1.044) | 0.42 | 0.988 | (0.904, | 1.079) | 0.79 |
|  | March | 0.902 | (0.840, | 0.970) | 0.0052 | 0.926 | (0.849, | 1.011) | 0.088 |
|  | April | 0.883 | (0.821, | 0.949) | 7.4e-04 | 0.887 | (0.812, | 0.969) | 0.0078 |
|  | May | 0.803 | (0.746, | 0.865) | 6.4e-09 | 0.823 | (0.753, | 0.900) | 1.8e-05 |
|  | June | 0.773 | (0.717, | 0.833) | 2.1e-11 | 0.809 | (0.739, | 0.885) | 3.9e-06 |
|  | July | 0.756 | (0.700, | 0.815) | 4.2e-13 | 0.748 | (0.682, | 0.819) | 4.7e-10 |
|  | August | 0.803 | (0.746, | 0.866) | 8.6e-09 | 0.824 | (0.753, | 0.901) | 2.1e-05 |
|  | September | 0.846 | (0.786, | 0.911) | 9.5e-06 | 0.892 | (0.817, | 0.973) | 0.010 |
|  | October | 0.850 | (0.790, | 0.915) | 1.4e-05 | 0.860 | (0.788, | 0.939) | 7.5e-04 |
|  | November | 0.829 | (0.771, | 0.893) | 6.2e-07 | 0.842 | (0.771, | 0.919) | 1.2e-04 |
|  | December | 0.926 | (0.864, | 0.993) | 0.030 | 0.961 | (0.884, | 1.043) | 0.34 |
| Day of the week of admission | Sunday | 1 |  |  |  | 1 |  |  |  |
|  | Monday | 0.974 | (0.919, | 1.032) | 0.37 | 0.995 | (0.929, | 1.066) | 0.89 |
|  | Tuesday | 0.943 | (0.889, | 1.000) | 0.050 | 0.970 | (0.905, | 1.040) | 0.40 |
|  | Wednesday | 0.943 | (0.889, | 1.000) | 0.049 | 0.953 | (0.889, | 1.023) | 0.18 |
|  | Thursday | 0.945 | (0.891, | 1.002) | 0.058 | 0.961 | (0.897, | 1.031) | 0.27 |
|  | Friday | 0.973 | (0.918, | 1.032) | 0.37 | 1.007 | (0.939, | 1.079) | 0.85 |
|  | Saturday | 0.972 | (0.916, | 1.032) | 0.35 | 0.977 | (0.910, | 1.049) | 0.52 |
| Weekend Admission | Week day | 1 |  |  |  | 1 |  |  |  |
|  | Weekend day | 1.032 | (1.000, | 1.064) | 0.052 | 1.025 | (0.988, | 1.064) | 0.19 |
| Out of hours admission | In hours | 1 |  |  |  | 1 |  |  |  |
|  | Out of hours | 1.110 | (1.076, | 1.145) | 3.7e-11 | 1.114 | (1.074, | 1.156) | 6.6e-09 |
| Day of the week of surgery | Sunday | 1 |  |  |  | 1 |  |  |  |
|  | Monday | 0.967 | (0.912, | 1.026) | 0.27 | 0.986 | (0.919, | 1.057) | 0.68 |
|  | Tuesday | 0.961 | (0.906, | 1.019) | 0.18 | 0.980 | (0.914, | 1.051) | 0.57 |
|  | Wednesday | 0.945 | (0.891, | 1.002) | 0.059 | 0.978 | (0.912, | 1.048) | 0.53 |
|  | Thursday | 0.969 | (0.914, | 1.028) | 0.30 | 0.962 | (0.897, | 1.032) | 0.28 |
|  | Friday | 0.938 | (0.884, | 0.995) | 0.032 | 0.948 | (0.883, | 1.017) | 0.13 |
|  | Saturday | 0.948 | (0.892, | 1.007) | 0.081 | 0.966 | (0.899, | 1.037) | 0.34 |
| Weekend Surgery | Week day | 1 |  |  |  | 1 |  |  |  |
|  | Weekend day | 0.999 | (0.969, | 1.031) | 0.97 | 0.993 | (0.957, | 1.030) | 0.71 |
| Out of hours surgery | In hours | 1 |  |  |  | 1 |  |  |  |
|  | Out of hours | 1.001 | (0.935, | 1.071) | 0.99 | 1.035 | (0.953, | 1.124) | 0.42 |
| Time to Surgery hours decimal |  | 1.002 | (1.002, | 1.003) | 0 | 1.003 | (1.002, | 1.003) | 0 |
| ln(Time to surgery) (Hours) |  | 1.163 | (1.136, | 1.191) | 0 | 1.180 | (1.146, | 1.214) | 0 |
| Time to surgery categorical | >=0 & <=24 | 1 |  |  |  | 1 |  |  |  |
|  | >24 & <=36 | 1.179 | (1.133, | 1.227) | 4.4e-16 | 1.185 | (1.132, | 1.241) | 6.1e-13 |
|  | >36 & <=48 | 1.155 | (1.101, | 1.212) | 4.6e-09 | 1.127 | (1.064, | 1.195) | 5.1e-05 |
|  | >48 & <=60 | 1.426 | (1.333, | 1.526) | 0 | 1.374 | (1.265, | 1.493) | 5.6e-14 |
|  | >60 & <=72 | 1.308 | (1.207, | 1.416) | 5.0e-11 | 1.352 | (1.227, | 1.489) | 1.1e-09 |
|  | >72 | 1.559 | (1.469, | 1.654) | 0 | 1.605 | (1.492, | 1.727) | 0 |
| Time to surgery categorical |  | 1.028 | (1.078, | 1.100) | 0 | 1.092 | (1.079, | 1.105) | 0 |
| Time to surgery <= 24 hours | <=24 hours | 1 |  |  |  | 1 |  |  |  |
|  | >24 hours | 1.252 | (1.214, | 1.292) | 0 | 1.245 | (1.200, | 1.291) | 0 |
| Time to surgery <= 36 hours | <=36 hours | 1 |  |  |  | 1 |  |  |  |
|  | >36 hours | 1.245 | (1.204, | 1.287) | 0 | 1.230 | (1.181, | 1.281) | 0 |
| Time to surgery <= 48 hours | <=48 hours | 1 |  |  |  | 1 |  |  |  |
|  | >48 hours | 1.359 | (1.306, | 1.415) | 0 | 1.372 | (1.306, | 1.442) | 0 |
| Time to surgery <= 60 hours | <=60 hours | 1 |  |  |  | 1 |  |  |  |
|  | >60 hours | 1.348 | (1.285, | 1.413) | 0 | 1.396 | (1.317, | 1.480) | 0 |
| Time to surgery <= 72 hours | <=72 hours | 1 |  |  |  | 1 |  |  |  |
|  | >72 hours | 1.423 | (1.344, | 1.506) | 0 | 1.474 | (1.373, | 1.582) | 0 |

Table S6: Model-1, Minimally Adjusted models, adjusted for pre-admission patient characteristics (Fracture type, ASA, AMTS, Pathology, Mobility) and their association with 30 day mortality after hip fracture; N(Multiple Imputation)=241446, N(Complete Cases)=182772 .

|  |  | Multiple Imputation | | | | Complete Cases | | | |
| --- | --- | --- | --- | --- | --- | --- | --- | --- | --- |
| Variable | Level | OR | 95% CI | | p= | OR | 95% CI | | p= |
| Year of admission -2011 | 0 | 1 |  |  |  | 1 |  |  |  |
|  | 1 | 1.004 | (0.959, | 1.050) | 0.88 | 1.076 | (1.014, | 1.143) | 0.015 |
|  | 2 | 0.919 | (0.878, | 0.961) | 2.6e-04 | 0.979 | (0.923, | 1.038) | 0.48 |
|  | 3 | 0.792 | (0.756, | 0.830) | 0 | 0.847 | (0.798, | 0.898) | 3.1e-08 |
| Month of admission | January | 1 |  |  |  | 1 |  |  |  |
|  | February | 0.977 | (0.906, | 1.053) | 0.54 | 1.004 | (0.916, | 1.100) | 0.93 |
|  | March | 0.902 | (0.837, | 0.972) | 0.0070 | 0.929 | (0.848, | 1.017) | 0.11 |
|  | April | 0.883 | (0.819, | 0.952) | 0.0011 | 0.891 | (0.813, | 0.976) | 0.013 |
|  | May | 0.803 | (0.743, | 0.866) | 1.8e-08 | 0.829 | (0.756, | 0.909) | 6.9e-05 |
|  | June | 0.763 | (0.705, | 0.825) | 1.1e-11 | 0.806 | (0.735, | 0.885) | 5.9e-06 |
|  | July | 0.757 | (0.700, | 0.819) | 3.5e-12 | 0.755 | (0.687, | 0.830) | 6.1e-09 |
|  | August | 0.796 | (0.737, | 0.860) | 7.4e-09 | 0.830 | (0.756, | 0.910) | 7.5e-05 |
|  | September | 0.849 | (0.786, | 0.916) | 2.6e-05 | 0.901 | (0.823, | 0.986) | 0.024 |
|  | October | 0.837 | (0.775, | 0.903) | 4.1e-06 | 0.855 | (0.781, | 0.936) | 6.8e-04 |
|  | November | 0.819 | (0.759, | 0.884) | 2.8e-07 | 0.839 | (0.766, | 0.919) | 1.5e-04 |
|  | December | 0.942 | (0.876, | 1.013) | 0.11 | 0.992 | (0.910, | 1.081) | 0.85 |
| Day of the week of admission | Sunday | 1 |  |  |  | 1 |  |  |  |
|  | Monday | 0.972 | (0.915, | 1.032) | 0.35 | 0.990 | (0.922, | 1.063) | 0.79 |
|  | Tuesday | 0.930 | (0.875, | 0.988) | 0.019 | 0.956 | (0.889, | 1.027) | 0.21 |
|  | Wednesday | 0.931 | (0.876, | 0.990) | 0.022 | 0.939 | (0.873, | 1.009) | 0.087 |
|  | Thursday | 0.942 | (0.886, | 1.001) | 0.054 | 0.957 | (0.891, | 1.029) | 0.23 |
|  | Friday | 0.983 | (0.925, | 1.044) | 0.57 | 1.021 | (0.950, | 1.096) | 0.58 |
|  | Saturday | 0.999 | (0.939, | 1.063) | 0.98 | 1.003 | (0.932, | 1.079) | 0.94 |
| Weekend Admission | Week day | 1 |  |  |  | 1 |  |  |  |
|  | Weekend day | 1.053 | (1.019, | 1.088) | 0.0020 | 1.050 | (1.010, | 1.091) | 0.013 |
| Out of hours admission | In hours | 1 |  |  |  | 1 |  |  |  |
|  | Out of hours | 1.020 | (0.987, | 1.053) | 0.23 | 1.017 | (0.979, | 1.056) | 0.38 |
| Day of the week of surgery | Sunday | 1 |  |  |  | 1 |  |  |  |
|  | Monday | 0.939 | (0.884, | 0.998) | 0.044 | 0.959 | (0.892, | 1.031) | 0.25 |
|  | Tuesday | 0.919 | (0.864, | 0.976) | 0.0062 | 0.936 | (0.871, | 1.006) | 0.073 |
|  | Wednesday | 0.896 | (0.842, | 0.952) | 4.1e-04 | 0.923 | (0.859, | 0.993) | 0.031 |
|  | Thursday | 0.918 | (0.864, | 0.976) | 0.0060 | 0.908 | (0.845, | 0.976) | 0.0091 |
|  | Friday | 0.888 | (0.835, | 0.944) | 1.4e-04 | 0.898 | (0.835, | 0.965) | 0.0036 |
|  | Saturday | 0.932 | (0.875, | 0.991) | 0.026 | 0.953 | (0.885, | 1.026) | 0.20 |
| Weekend Surgery | Week day | 1 |  |  |  | 1 |  |  |  |
|  | Weekend day | 1.019 | (0.987, | 1.053) | 0.25 | 1.016 | (0.977, | 1.055) | 0.43 |
| Out of hours surgery | In hours | 1 |  |  |  | 1 |  |  |  |
|  | Out of hours | 1.026 | (0.956, | 1.101) | 0.47 | 1.055 | (0.969, | 1.149) | 0.22 |
| Time to Surgery (hours) |  | 1.000 | (1.000, | 1.001) | 0.37 | 1.000 | (1.000, | 1.001) | 0.086 |
| ln(Time to surgery) (hour) |  | 1.043 | (1.019, | 1.068) | 4.3e-04 | 1.055 | (1.025, | 1.086) | 2.9e-04 |
| Time to surgery categorical | >=0 & <=24 | 1 |  |  |  | 1 |  |  |  |
|  | >24 & <=36 | 1.078 | (1.035, | 1.124) | 3.2e-04 | 1.081 | (1.031, | 1.134) | 0.0013 |
|  | >36 & <=48 | 1.073 | (1.021, | 1.128) | 0.0058 | 1.046 | (0.985, | 1.111) | 0.14 |
|  | >48 & <=60 | 1.200 | (1.118, | 1.287) | 4.2e-07 | 1.140 | (1.046, | 1.243) | 0.0028 |
|  | >60 & <=72 | 1.108 | (1.019, | 1.204) | 0.016 | 1.143 | (1.033, | 1.264) | 0.0094 |
|  | >72 | 1.118 | (1.051, | 1.191) | 4.6e-04 | 1.145 | (1.060, | 1.237) | 5.9e-04 |
| Time to surgery categorical |  | 1.028 | (1.018, | 1.039) | 1.8e-07 | 1.030 | (1.017, | 1.043) | 6.4e-06 |
| Time to surgery <= 24 hours | <=24 hours | 1 |  |  |  | 1 |  |  |  |
|  | >24 hours | 1.097 | (1.062, | 1.133) | 2.4e-08 | 1.090 | (1.050, | 1.133) | 8.7e-06 |
| Time to surgery <= 36 hours | <=36 hours | 1 |  |  |  | 1 |  |  |  |
|  | >36 hours | 1.084 | (1.047, | 1.123) | 6.6e-06 | 1.071 | (1.026, | 1.117) | 0.0015 |
| Time to surgery <= 48 hours | <=48 hours | 1 |  |  |  | 1 |  |  |  |
|  | >48 hours | 1.105 | (1.059, | 1.153) | 4.0e-06 | 1.111 | (1.054, | 1.170) | 7.6e-05 |
| Time to surgery <= 60 hours | <=60 hours | 1 |  |  |  | 1 |  |  |  |
|  | >60 hours | 1.068 | (1.015, | 1.122) | 0.011 | 1.104 | (1.038, | 1.174) | 0.0016 |
| Time to surgery <= 72 hours | <=72 hours | 1 |  |  |  | 1 |  |  |  |
|  | >72 hours | 1.067 | (1.005, | 1.134) | 0.033 | 1.099 | (1.020, | 1.184) | 0.013 |

Table S7: Model-2, Multivariate adjusted models, adjusted for all variables and pre-admission patient characteristics (Fracture type, ASA, AMTS, Pathology, Mobility) and their association with 30 day mortality after hip fracture. N(Multiple Imputation)=241446, N(Complete Cases)=182772.

|  |  | Multiple Imputation | | | | Complete Cases | | | |
| --- | --- | --- | --- | --- | --- | --- | --- | --- | --- |
| Variable | Level | OR | (95%CI) | | p= | OR | (95%CI) | | p= |
| Year of admission -2011 | 0 | 1 |  |  |  | 1 |  |  |  |
|  | 1 | 1.007 | (0.962, | 1.053) | 0.78 | 1.083 | (1.021, | 1.150) | 0.0085 |
|  | 2 | 0.922 | (0.881, | 0.965) | 5.1e-04 | 0.983 | (0.927, | 1.043) | 0.57 |
|  | 3 | 0.795 | (0.758, | 0.833) | 0 | 0.849 | (0.800, | 0.900) | 5.1e-08 |
| Month of admission | January | 1 |  |  |  | 1 |  |  |  |
|  | February | 0.976 | (0.905, | 1.052) | 0.52 | 1.001 | (0.914, | 1.097) | 0.98 |
|  | March | 0.898 | (0.834, | 0.968) | 0.0051 | 0.923 | (0.843, | 1.010) | 0.083 |
|  | April | 0.879 | (0.815, | 0.948) | 7.9e-04 | 0.885 | (0.807, | 0.969) | 0.0085 |
|  | May | 0.798 | (0.739, | 0.861) | 7.3e-09 | 0.820 | (0.748, | 0.900) | 2.6e-05 |
|  | June | 0.757 | (0.700, | 0.819) | 3.2e-12 | 0.796 | (0.725, | 0.874) | 1.6e-06 |
|  | July | 0.753 | (0.696, | 0.814) | 1.3e-12 | 0.746 | (0.678, | 0.820) | 1.3e-09 |
|  | August | 0.792 | (0.733, | 0.856) | 3.4e-09 | 0.820 | (0.748, | 0.900) | 2.7e-05 |
|  | September | 0.842 | (0.780, | 0.910) | 1.2e-05 | 0.888 | (0.811, | 0.973) | 0.010 |
|  | October | 0.834 | (0.773, | 0.900) | 3.0e-06 | 0.847 | (0.774, | 0.928) | 3.3e-04 |
|  | November | 0.815 | (0.755, | 0.879) | 1.4e-07 | 0.830 | (0.758, | 0.908) | 5.4e-05 |
|  | December | 0.940 | (0.874, | 1.010) | 0.091 | 0.982 | (0.901, | 1.070) | 0.67 |
| Day of the week of admission | Sunday | 1 |  |  |  | 1 |  |  |  |
|  | Monday | 0.986 | (0.919, | 1.058) | 0.70 | 1.014 | (0.931, | 1.103) | 0.75 |
|  | Tuesday | 0.933 | (0.862, | 1.008) | 0.080 | 0.978 | (0.889, | 1.075) | 0.64 |
|  | Wednesday | 0.915 | (0.844, | 0.992) | 0.032 | 0.967 | (0.876, | 1.066) | 0.50 |
|  | Thursday | 0.941 | (0.868, | 1.020) | 0.14 | 0.994 | (0.902, | 1.096) | 0.90 |
|  | Friday | 0.951 | (0.881, | 1.027) | 0.20 | 1.020 | (0.930, | 1.118) | 0.67 |
|  | Saturday | 0.948 | (0.884, | 1.017) | 0.14 | 0.969 | (0.891, | 1.055) | 0.47 |
| Day of the week of surgery | Sunday | 1 |  |  |  | 1 |  |  |  |
|  | Monday | 0.908 | (0.847, | 0.974) | 0.0067 | 0.943 | (0.868, | 1.025) | 0.17 |
|  | Tuesday | 0.894 | (0.827, | 0.966) | 0.0048 | 0.918 | (0.835, | 1.008) | 0.074 |
|  | Wednesday | 0.902 | (0.832, | 0.979) | 0.013 | 0.923 | (0.836, | 1.019) | 0.11 |
|  | Thursday | 0.942 | (0.869, | 1.022) | 0.15 | 0.917 | (0.831, | 1.013) | 0.087 |
|  | Friday | 0.900 | (0.833, | 0.974) | 0.0086 | 0.892 | (0.812, | 0.981) | 0.018 |
|  | Saturday | 0.938 | (0.874, | 1.008) | 0.079 | 0.932 | (0.855, | 1.015) | 0.11 |
| Out of hours surgery | In hours | 1 |  |  |  | 1 |  |  |  |
|  | Out of hours | 1.009 | (0.940, | 1.083) | 0.81 | 1.041 | (0.955, | 1.133) | 0.36 |
| Time to surgery <= 24 hours | <=24 hours | 1 |  |  |  | 1 |  |  |  |
|  | >24 hours | 1.095 | (1.060, | 1.131) | 5.2e-08 | 1.090 | (1.049, | 1.132) | 1.0e-05 |

The baseline level of each variable is indicated. i.e. the model baseline is year=0, month=January, day of the week of admission=Sunday, day of the week of surgery=Sunday, out of hours surgery = in hours, time to surgery= within 24 hours.

Table S8: Multivariate adjusted models investigating the association between time of surgery and mortality at 30 days in patients with complete covariate information (Complete Cases). Models progressively adjusted for non-surgical interventions (Model-3) N(Complete Cases)= 178551, Surgical treatments (Model-4) N(Complete Cases)= 157557, Index of Multiple Deprivation (Model-5) N(Complete Cases)= 149966.

|  |  | Model-3 | | | | Model-4 | | | | Model5 | | | |
| --- | --- | --- | --- | --- | --- | --- | --- | --- | --- | --- | --- | --- | --- |
|  |  | OR | (95%CI) | | p= | OR | (95%CI) | | p= | OR | (95%CI) | | p= |
| Month of Year | January | 1 |  |  |  | 1 |  |  |  | 1 |  |  |  |
|  | February | 1.016 | (0.925, | 1.116) | 0.74 | 1.017 | (0.915, | 1.129) | 0.76 | 1.003 | (0.901, | 1.117) | 0.95 |
|  | March | 0.946 | (0.862, | 1.039) | 0.25 | 0.921 | (0.830, | 1.023) | 0.12 | 0.917 | (0.824, | 1.020) | 0.11 |
|  | April | 0.889 | (0.810, | 0.976) | 0.014 | 0.882 | (0.797, | 0.977) | 0.016 | 0.877 | (0.791, | 0.974) | 0.014 |
|  | May | 0.828 | (0.753, | 0.910) | 9.2e-05 | 0.834 | (0.753, | 0.925) | 5.5e-04 | 0.831 | (0.748, | 0.923) | 5.3e-04 |
|  | June | 0.810 | (0.736, | 0.891) | 1.5e-05 | 0.821 | (0.740, | 0.911) | 1.9e-04 | 0.820 | (0.738, | 0.911) | 2.3e-04 |
|  | July | 0.761 | (0.690, | 0.838) | 3.0e-08 | 0.766 | (0.690, | 0.851) | 6.3e-07 | 0.775 | (0.696, | 0.863) | 3.1e-06 |
|  | August | 0.825 | (0.751, | 0.908) | 7.4e-05 | 0.838 | (0.756, | 0.929) | 8.0e-04 | 0.845 | (0.760, | 0.938) | 0.0017 |
|  | September | 0.908 | (0.828, | 0.996) | 0.040 | 0.889 | (0.804, | 0.984) | 0.023 | 0.888 | (0.801, | 0.984) | 0.024 |
|  | October | 0.867 | (0.790, | 0.951) | 0.0024 | 0.855 | (0.773, | 0.946) | 0.0024 | 0.857 | (0.773, | 0.950) | 0.0034 |
|  | November | 0.856 | (0.781, | 0.939) | 0.0010 | 0.858 | (0.776, | 0.949) | 0.0029 | 0.855 | (0.772, | 0.948) | 0.0030 |
|  | December | 1.004 | (0.920, | 1.096) | 0.93 | 1.004 | (0.912, | 1.105) | 0.94 | 1.001 | (0.907, | 1.104) | 0.99 |
| Sunday surgery | MTWTFS surg | 1 |  |  |  | 1 |  |  |  | 1 |  |  |  |
|  | Sunday surg | 1.069 | (1.009, | 1.133) | 0.025 | 1.060 | (0.997, | 1.128) | 0.062 | 1.060 | (0.995, | 1.129) | 0.072 |
| Out of hours surgery | In hours | 1 |  |  |  | 1 |  |  |  | 1 |  |  |  |
|  | Out of hours | 1.039 | (0.953, | 1.134) | 0.38 | 1.043 | (0.951, | 1.143) | 0.38 | 1.067 | (0.971, | 1.171) | 0.18 |
| Time to surgery <= 24 hours | <=24 hours | 1 |  |  |  | 1 |  |  |  | 1 |  |  |  |
|  | >24 hours | 1.078 | (1.037, | 1.121) | 1.5e-04 | 1.088 | (1.044, | 1.134) | 7.2e-05 | 1.115 | (1.068, | 1.163) | 5.7e-07 |

Model-3 = Pre admission characteristics + Non-surgical interventions, Model-4 = Model-3 + Surgical Treatments, Model-5 = Model-4 + Index of Multiple Deprivation. (Preadmission Characteristics = Fracture type, ASA, AMTS, Pathology, Mobility; Non-surgical interventions= Falls Assessment, MDT meeting; Surgical interventions = anaesthetic type, operation type; Index of Multiple Deprivation=Index of Multiple Deprivation Older People England, Index of Multiple Deprivation Older People Wales).

Table S9: Sensitivity analysis exploring the effect of seasonal adjustment on multivariate adjusted models investigating the association between time of surgery and mortality at 30 days. Models adjusted for patient characteristics, non-surgical treatments, and surgical treatment. Model-7 uses an elapsed month seasonal specification, whereas Model-8 uses trigonometric regression approach (Fourier series) to Model-the effect of season. N(Multiple Imputation)=241446, N(Complete Cases)= 157557.

|  |  | Multiple Imputation | | | | Complete Cases | | | |
| --- | --- | --- | --- | --- | --- | --- | --- | --- | --- |
|  |  | Model-7 | | | | Model-8 | | | |
| Variable | Level | OR | (95%CI) | | p= | OR | (95%CI) | | p= |
| Sunday surgery | MTWTFS surg | 1 |  |  |  | 1 |  |  |  |
|  | Sunday surg | 1.083 | (1.032, | 1.136) | 0.0012 | 1.059 | (0.996, | 1.127) | 0.067 |
| Out of hours surgery | In hours | 1 |  |  |  | 1 |  |  |  |
|  | Out of hours | 1.011 | (0.941, | 1.086) | 0.76 | 1.044 | (0.952, | 1.145) | 0.36 |
| Time to surgery <= 24 hours | <=24 hours | 1 |  |  |  | 1 |  |  |  |
|  | >24 hours | 1.089 | (1.054, | 1.126) | 3.5e-07 | 1.089 | (1.045, | 1.136) | 5.6e-05 |
| Sunday surgery | MTWTFS surg | 1 |  |  |  | 1 |  |  |  |
|  | Sunday surg | 1.083 | (1.032, | 1.136) | 0.0011 | 1.061 | (0.998, | 1.129) | 0.059 |
| Out of hours surgery | In hours | 1 |  |  |  | 1 |  |  |  |
|  | Out of hours | 1.011 | (0.941, | 1.086) | 0.76 | 1.042 | (0.950, | 1.143) | 0.38 |
| Time to surgery <= 24 hours | <=24 hours | 1 |  |  |  | 1 |  |  |  |
|  | >24 hours | 1.089 | (1.054, | 1.125) | 4.0e-07 | 1.088 | (1.044, | 1.134) | 6.6e-05 |
